# Supplementary material for: WordCommentsAnalyzer: A windows software tool for qualitative research
Source: F1000Res. 2018 Sep 4;7:536. Originally published 2018 May 3. [Version 2] doi: 10.12688/f1000research.14819.2 (PMC6137411; doi:10.12688/f1000research.14819.2)
Supplement: Supplementary file 3 [file f1000research-7-17614-s0002.tgz › b2a79496-4ada-47c4-9682-a99ce867836d.zip!/Eight Iranian Health Journals/The Journal of Toloo-e-Behdasht_33.docx]

© The Journal of Toloo-e-Behdasht. This work is licensed under CC BY 4.0 (https://creativecommons.org/licenses/by/4.0/).

Volume & Issue: دوره 11 - شماره 3

Title: بررسی پتانسیل خوردگی و رسوب گذاری آب شرب شبکه توزیع شهر کرمان

http://tbj.ssu.ac.ir/article-1-320-fa.html

سابقه و اهداف: وقوع پدیده های خوردگی و رسوب گذاری درشبکه آب آشامیدنی باعث آسیب های اقتصادی و بهداشتی می گردد. هدف این مطالعه تعیین پتانسیل خوردگی و رسوب گذاری آب شرب شهر کرمان می باشد .   روش بررسی: مطالعه توصیفی- مقطعی است که در بازه زمانی مهر لغایت آذر سال 1390 انجام گرفت. جهت تعیین کیفیت شیمیایی آب در شبکه مورد نظر نمونه ها لحظه ای و به تعداد 40 نمونه برداشت و آنالیز شد. حجم نمونه های برداشتی4-2 لیتر و ظروف مورد استفاده جهت جمع آوری نمونه ها از نوع شیشه ای بود.کیفیت فیزیکی وشیمیایی نمونه های مورد بررسی و وضعیت پتانسیل خوردگی و رسوبگذاری با استفاده از اندیس اشباع لانژلیه ( LSI )، اندیس رایزنار ( RI ) پورکوریوس ( PSI ) و شاخص تهاجم ( LS ) تعیین گردید. آزمایشات بر اساس روشهای مندرج در کتاب استاندارد روشهای آزمایش آب و فاضلاب انجام گردید. داده ها با استفاده از آمار توصیفی تحلیل گردید.   یافته ها: نتایج آنالیزهای شیمیایی آب شرب مورد استفاده با مقادیر رهنمودی سازمان جهانی بهداشت و استاندارد های ایران مطابقت داشت. بیش از92 درصد نمونه هاشاخص لانژلیه بیش از صفر داشت. در بیش از82 درصد نمونه هاشاخص رایزنارکمتر از هفت بود. بر اساس شاخص پورکوریوس تنها 60 درصد نمونه ها رسوب گذار بود و اندیس تهاجم حکایت ازرسوب گذار بودن 90 در صد نمونه ها داشت. لذا با توجه به شاخص های LSI ، RI ، PSI و LS ، آب آشامیدنی در شبکه توزیع شهر کرمان دارای پتانسیل رسوبگذاری است.   نتیجه گیری: کیفیت شیمیایی آب شبکه شرب شهرکرمان از نظر شاخص های رسوب گذاری نامتعادل است و بیان می نماید که جریان آب در شبکه توزیع آب شهر کرمان باعث رسوبگذاری در شبکه توزیع آب شهر و سایر تاسیسات می گردد. از این رو لازم است برنامه ریزی مناسبی انجام گیرد تا از آسیب های اقتصادی جلوگیری شود.   خوردگی، رسوبگذاری، شبکه توزیع، کیفیت آب، شهر کرمان

Title: بررسی وضعیت خشونت خانگی در زنان متأهل مراجعه کننده به مراکز پزشکی قانونی و بهزیستی شهرستان یزد در سال 1389

http://tbj.ssu.ac.ir/article-1-321-fa.html

سابقه و اهداف: خشونت نسبت به زنان در خانواده، واقعیتی آزار دهنده است. این نوع خشونت، عرصه زندگی را برای زن و شوهر تنگ می‌کند و اثرهای متعدد و قابل توجهی بر کودکان و نوجوانان شاهد این خشونت بر جای می‌گذارد.   تحقیقات نشان می دهد در خانواده‌هایی که فرزندان کتک خوردن مادران خود را تجربه کرده‌اند، در بزرگسالی، دختران تمایل به تحمل همسرآزاری دارند و پسران، کتک زدن همسر را حق قانونی خود می‌دانند.   نتایج حاصل از طرح ملی بررسی خشونت خانگی علیه زنان در 28 استان که درمحدوده‌ زمانی سال 80 ـ 75 انجام شد حاکی از افزایش 23 درصدی خشونت خانگی علیه زنان در شهرستان یزد است تحقیق حاضر درصدد بررسی عوامل مؤثر بر خشونت خانگی علیه زنان در شهرستان یزد انجام گرفته است.   روش بررسی : روش مورد استفاده در این پژوهش از نوع پیمایش است. جامعه آماری تحقیق را کلیه زنان متأهلی که به علت همسرآزاری در 3 ماهه آخر سال 1389 به مراکز پزشکی قانونی و بهزیستی شهرستان یزد مراجعه نموده‌اند تشکیل می‌دهند. نمونه مورد بررسی 142 زن مورد خشونت قرار گرفته بوده و روش نمونه‌گیری به صورت نمونه در دسترس بوده است. ابزار گردآوری داده‌ها پرسش‌نامه بود و جهت تجزیه و تحلیل اطلاعات از دو روش آمار توصیفی و آمار استنباطی بر حسب مورد استفاده شده است.   یافته‌ها : یافته‌های پژوهش نشان می‌دهد که تفاوت سنی زوجین ، اعتقاد به باورهای مرد سالارانه و وضعیت زندگی شوهر در دوران کودکی از نظر مشاهده، تجربه و یادگیری خشونت در خانواده با خشونت علیه زنان ارتباط معنادار دارد.   نتیجه‌گیری : مهم‌ ترین نتیجه پژوهش تأکید بر نقش پررنگ تربیتی ـ فرهنگی در بروز پدیده همسرآزاری است. بطوریکه مردانی که در کودکی در خانواده شاهد کتک خوردن مادران خود بوده‌اند بیشتر به خشونت علیه زنان روی می‌آورند و زنانی که به باورهای مردسالارانه معتقدند و به نوعی آن را پذیرفته‌‌اند نیز بیشتر مورد خشونت واقع شده‌اند.

Title: بررسی پیشگویی کننده های مصرف سیگار براساس الگوی تصورات – تمایلات در دانش آموزان دبیرستانی شهر مراغه

http://tbj.ssu.ac.ir/article-1-322-fa.html

سابقه و اهداف : با توجه به ترکیب جمعیتی جوان کشور ایران و شیوع مصرف سیگار در میان جوانان و اهمیت شناخت عوامل تعیین کننده و پیش بینی کننده رفتار استعمال سیگار در اتخاذ و اجرای برنامه های پیشگیرانه، این مطالعه با هدف تعیین پیش بینی کننده های مصرف سیگار براساس الگوی تصورات- تمایلات در دانش آموزان دبیرستانی شهر مراغه انجام گرفت.   روش بررسی : در این مطالعه توصیفی همبستگی که به روش مقطعی انجام شد، 240 دانش آموز از طریق نمونه گیری تصادفی دو مرحله ای از 2 دبیرستان پسرانه و 2 دبیرستان دخترانه شهر مراغه در تحقیق وارد شدند. ابزار جمع آوری اطلاعات، پرسشنامه ای محقق ساخته با روائی و پایایی قابل قبول می باشد که شامل اطلاعات دموگرافیک و سازه های الگوی تصورات- تمایلات بود که به صورت خودگزارشی تکمیل می گردید. تجزیه و تحلیل داده ها با کمک نرم افزار آماری 16 SPSS وآزمون های آماری ضریب همبستگی ونیز آنالیز رگرسیون انجام شد.   یافته ها : آزمودنیها به ترتیب 3/77، 4/ 91، 7/80 ، 4/33 ،4/ 41 درصد میانگین از ماکزیمم نمره سازه های قصد عدم مصرف سیگار، هنجارهای اجتماعی، نگرش، تمایلات و تصورات راکسب نمودند. میانگین نمره سازه های تمایل، نگرش منفی به سیگار و تصورات مثبت به سیگار برحسب مصرف سیگار در افراد مورد مطالعه دارای اختلاف معنادار آماری بود. قصدعدم مصرف سیگار با تمایلات و تصورات مثبت نسبت به مصرف سیگار همبستگی منفی و با هنجارهای اجتماعی نسبت به قصد عدم مصرف سیگار همبستگی مثبت داشت. همچنین یافته ها نشان دادکه متغیرتمایل بیشتر از قصد (73/1= Odd Ratio در مقابل 10/1)احتمال تجربه مصرف سیگار را افزایش می دهد.   نتیجه گیری : افراد با توجه به شرایط زندگی ومحیط اجتماعی، امکان دارد تصوراتی مثبت یا منفی از فرد سیگاری داشته باشند و این تصورات می تواند در بوجود آمدن تمایل به مصرف سیگار موثر باشند و این ضرورت مداخلات خاص که تصورات و تمایلات را مورد هدف قرار دهد را می طلبد.

Title: بررسی پتانسیل اقتصادی و زیست محیطی ناشی از طرح تفکیک از مبدأ پسماندهای جامد شهری در نواحی هفت گانه منطقه 5 شهر تهران

http://tbj.ssu.ac.ir/article-1-323-fa.html

سابقه و اهداف: منطقه 5 شهرداری تهران به لحاظ وسعت، جمعیت، موقعیت جغرافیائی و تعداد نواحی از مناطق بسیار بزرگ شهر تهران می باشد. این منطقه از ظرفیت های مناسب جهت اجرای طرح تفکیک از مبدأ پسماندهای جامد شهری برخور دار است. در این پژوهش، میزان پتانسیل اقتصادی و زیست محیطی حاصل از انجام طرخ تفکیک از مبداء پسماندهای جامد منطقه 5 شهر تهران مورد بررسی قرار گرفته است.   روش بررسی: این پژوهش یک مطالعه توصیفی مقطعی بوده که در سال 1390 با جمع آوری اطلاعات از طریق مصاحبه حضوری با مسئولین بازیافت منطقه 5 شهر تهران و بررسی اسناد و مدارک موجود در نواحی هفت گانه و نیز با کمک مطالعات کتابخانه ای انجام گردیده است. داده های حاصل از این مطالعه با استفاده از نرم افزار آماری16 SPSS مورد تجزیه و تحلیل قرار گرفت.   یافته ها: از میزان 4241 تن بازیافت ماهیانه پسماند جامد، 1/32 درصد(1361 تن) آن را کاغذ و مقوا تشکیل می دهد. همچنین میزان بازیافت اجزای نان خشک، پلاستیک و پت، زایدات آهنی، شیشه و منسوجات به ترتیب 7/13، 16، 5، 2/6 و 3/4 درصد بدست آمد. با توجه به میزان بازیافت سالیانه کاغذ و مقوا(16332 تن) از لحاظ اقتصادی سود حاصل از فروش کاغذ 3266400000 ریال، سود ناشی از کاهش قطع درختان 2345490000 ریال، سود حاصل از کاهش مصرف انرژی و آب به ترتیب 8201547648 ریال و 9933840 ریال و میزان کاهش هزینه های جمع آوری، حمل و دفع پسماند 928998000 ریال محاسبه گردید.   نتیجه گیری: با عنایت به نتایج این پژوهش، طرح تفکیک و بازیافت پسماندهای جامد خشک در منطقه 5 شهرداری تهران می تواند بسیاری از مشکلات اقتصادی و زیست محیطی موجود در این منطقه را مرتفع نماید.

Title: تاثیر مداخله آموزشی به منظور ارتقاء استفاده از کمربند ایمنی مبتنی بر مدل اعتقاد بهداشتی در بین رانندگان خودروهای سواری

http://tbj.ssu.ac.ir/article-1-324-fa.html

سابقه و اهداف: حوادث ترافیکی در ایران همواره بیشترین میزان مرگ و میر را به دنبال داشته است و یکی از مهم‌ترین علل مرگ و میرها در حین تصادفات رانندگی نبستن کمربند ایمنی می‌باشد، بطوریکه 63 درصد از مرگ‌ها را به آن نسبت می‌دهند. شواهد محکمی در مورد موثر بودن رویکرد استفاده از کمربند ایمنی در کاهش شدت بروز حوادث ترافیکی وجود دارد. ارائه برنامه‌های آموزشی مبتنی بر مبانی تئوریک مهمترین محور برنامه‌های آموزش بهداشت است. هدف از انجام مطالعه حاضر بررسی تاثیر اجرای برنامه آموزشی مبتنی بر مدل اعتقاد بهداشتی به منظور ارتقای استفاده از کمربند ایمنی در بین رانندگان بود.   روش بررسی: این پژوهش یک مطالعه مداخله‌ای نیمه تجربی بود که در بین کارکنان مراکز بهداشت شهرستان اهواز که دارای خودروی سواری بودند، انجام گرفت. بدین منظور به طور تصادفی از مرکز بهداشت شرق 80 نفر در گروه مداخله و مرکز بهداشت غرب 73 نفر در گروه شاهد وارد مطالعه شدند و به مدت دو ماه مورد پیگیری قرار گرفتند. پس از انجام ارزشیابی تشخیصی اقدام به مداخله آموزشی گردید. داده های حاصل از این پژوهش با استفاده از پرسشنامه محقق ساز، که پایایی آن با انجام یک مطالعه پایلوت توسط آزمون ضریب آلفا کرونباخ و روایی آن نیز با نظر کارشناسان مربوطه حاصل شده بود، به روش خودگزارش دهی، جمع آوری و سپس توسط نرم افزار آماری SPSS ویرایش هفدهم مورد تجزیه و تحلیل قرار گرفتند.   یافته ها: قبل از مداخله دو گروه از نظر متغیرهای زمینه‌ای یکسان بودند و بین سازه‌های مدل در دو گروه اختلاف معناداری مشاهده نشد. ولی بعد از اجرای برنامه آموزشی اختلاف معناداری برای افزایش میانگین نمره سازه‌های مدل اعتقاد بهداشتی و رفتار استفاده از کمربند ایمنی در بین گروه مداخله وجود داشت (05/0 > P )، اما در گروه شاهد اینگونه نبود. همچنین ارتباط معناداری بین بهره گیری از برنامه آموزشی و سازه راهنما برای عمل در بین هر دو گروه مشاهده شد.   نتیجه گیری: نتایج نشان دهنده ارتقاء سازه‌های مدل اعتقاد بهداشتی و رفتار در استفاده از کمربند ایمنی بعد از اتمام برنامه آموزشی در بین گروه مداخله می‌باشد که نشان از موثر بودن آموزش‌های مبتنی بر مدل‌ اعتقاد بهداشتی می باشد.

Title: تأثیر برنامه آموزش و مشاوره قبل از ازدواج بر آگاهی و نگرش زوجین نسبت به بهداشت باروری

http://tbj.ssu.ac.ir/article-1-325-fa.html

سابقه و اهداف: با آغاز زندگی مشترک، زوجین نیازمند اطلاعات مناسب و دقیق در زمینه جنبه های گوناگون بهداشت باروری می باشند.لذا این مطالعه به منظور تعیین تاثیر برنامه آموزش و مشاوره قبل از ازدواج بر آگاهی و نگرش زوجین نسبت به بهداشت باروری طراحی و اجرا گردید.   روش بررسی: دراین مطالعه نیمه تجربی ، تعداد 250 زوج شرکت کننده در کلاسهای مشاوره قبل از ازدواج مرکز مشاوره سلامت شهرستان زابل در سال 1388 با استفاده از روش نمونه گیری غیر احتما لی آسان ا نتخاب و مورد مطالعه قرار گرفتند.ابزار جمع آوری داده ها پرسشنامه خود ساخته شامل سه بخش اطلاعات فردی ،آگاهی و نگرش بود که در قبل و بعد از آموزش تکمیل گردید . برای انجام مداخله و برنامه آموزشی از کارشناسان بهداشت عمومی استفاده گردید.داده ها پس از جمع آوری با استفاده از آمار توصیفی و آزمونهای آماری t زوج شده و t مستقل ،آنالیز واریانس یک طرفه و X2 مورد تجزیه قرار گرفت .   یافته ها :میانگین سنی افراد مورد مطالعه 899/3 ± 78/21 سال بود. میانگین نمره آگاهی و نگرش افراد مورد مطالعه به ترتیب در قبل و بعد از آموزش از 44/2 ± 85/5 به 87/2 ± 92/8 واز 75/1 ± 25/10 به 71 /1 ± 12/11 افزایش پیدا کرد(05/0 ≥ p ). مقایسه تغییرات آگاهی و نگرش در قبل و بعد از آموزش افراد مورد مطالعه بر حسب هریک از متغیر های جنس،شغل و سطح تحصیلات.اختلاف آماری معنی داری را نشان داد(05/0 ≥ p ).   نتیجه گیری: هر چند برنامه آموزش و مشاوره قبل از ازدواج نسبتا بر آگاهی و نگرش زوجین تاثیر دارد ولی این برنامه ها نیاز به اصلاح اساسی دارد و مهمترین اصل در ارتقای کیفیت این برنامه ها انتخاب یک محتوی آموزشی فراگیر بر اساس نیازسنجی آموزشی و مشکلات اساسی زوجین قبل از ازدواج و اختصاص زمان بیشتری به این کلاسها می باشد .

Title: بررسی مقایسه ای عملکرد حذف 2-کلروفنل و 4-کلروفنل توسط کربن فعال گرانوله و خاکستر سبوس برنج

http://tbj.ssu.ac.ir/article-1-326-fa.html

سابقه و اهداف: آلودگی فاضلاب­ها بدلیل حضور آلاینده­های مختلف نظیر رنگ­ها، هیدروکربن­های هالوژنه، ترکیبات فنلی و غیره می­باشد. خاکسترسبوس برنج یک جاذب ارزان قیمت، جدید و با تولید قابل­توجه در کشور است و هدف این مطالعه مقایسه کارایی جاذب ارزان قیمت خاکستر سبوس برنج و گران قیمت کربن فعال گرانوله در حذف 2-کلروفنل و 4-کلروفنل از محیط­های آبی است.   روش بررسی: آزمایشات بصورت ناپیوسته با بررسی تاثیر فاکتورهایی نظیر pH ، زمان تماس، غلظت اولیه ترکیب فنلی و دوز جاذب روی جذب انجام شد. سنجش 2-کلروفنل و 4-کلروفنل توسط اسپکتروفتومتری، در طول موج­های 274 و 280 نانومتر انجام گرفت. در نهایت آنالیز داده­ها با استفاده از مدلهای ایزوترم و سینتیک صورت پذیرفت.   یافته­ها: حذف هردو ترکیب توسط کربن فعال بیشتر بود اما خاکستر سبوس برنج هم حذف قابل قبولی نشان داد. pH بهینه حذف دو ترکیب روی خاکستر سبوس برنج 5 و کربن فعال 7 بدست آمد. جذب با افزایش غلظت آلاینده و کاهش دوز جاذب کاهش یافت. مطالعات سینتیک جذب، تبعیت از مدل شبه درجه دوم روی هردو جاذب را آشکار ساخت. داده­های ایزوترم نیز نشان داد جذب این ترکیبات روی خاکستر سبوس برنج با مدل فروندلیچ و روی کربن فعال برای 2-کلروفنل با مدل لانگمویر و 4-کلروفنل با مدل فروندلیچ سازگار است.   نتیجه­گیری : هر دو جاذب کربن فعال و خاکستر سبوس برنج ظرفیت جذب مناسب داشتند و می‌توان با بهینه سازی فاکتور های موثر بر جذب، از خاکستر سبوس برنج به­عنوان جایگزینی کارامد و ارزان­قیمت در حذف این ترکیبات فنلی استفاده کرد.

Title: بررسی کارایی نانوتیوب‌های کربنی چند‌جداره در جذب اسید هیومیک در شرایط اسیدی از محلول‌های آبی

http://tbj.ssu.ac.ir/article-1-328-fa.html

سابقه و ا هداف: وجود اسید هیومیک به عنوان یکی از پیش‌سازهای تری هالومتان‌ها در منابع آب از مشکلات بهداشتی بسیاری از جوامع است. در همین راستا در سال‌های اخیر، روش‌های مختلفی برای حذف اسید هیومیک، مورد توجه قرار گرفته است . این مطالعه با هدف بررسی امکان‌سنجی استفاده از نانوتیوب‌های کربنی چند‌جداره به عنوان جاذب در فرآیند جذب اسید هیومیک در شرایط اسیدی از محلول‌های آبی انجام شده است .   روش بررسی : این مطالعه تجربی بصورت ناپیوسته انجام شده که در آن اثر پارامترهای مختلف زمان تماس، غلظت اسید هیومیک و میزان نانوتیوب در حذف اسید هیومیک بررسی شده است. در طی مراحل مختلف تحقیق، غلظت اسید هیومیک با استفاده از اسپکتروفتومتر در طول موج 254 نانومتر تعیین شد.   یافته ها: نتایج مطالعه نشان داد که حداکثر ظرفیت جذب سطحی اسید هیومیک بر روی نانوتیوب‌های کربنی چند‌جداره 37/31 میلی‌گرم بر گرم در شرایط pH اسیدی(4= pH ) اتفاق افتاده است. همچنین مشخص گردید که زمان مناسب جهت دستیابی به حداکثر جذب اسید هیومیک و حالت تعادل، 3 ساعت می‌باشد. با افزایش دوز جاذب از 04/0 به 08/0 گرم در 100 میلی‌لیتر راندمان حذف برای محلول اسید هیومیک با غلظت اولیه 20 میلی‌گرم در لیتر از 5/93 به 8/96 درصد افزایش می‌یابد. با کاهش غلظت اولیه‌ اسید هیومیک از 30 به 5 میلی‌گرم در لیتر در 4= pH و زمان 3 ساعت راندمان حذف از 5/91 به 9/96 درصد افزایش یافته است.   نتیجه‌گیری : به دلیل اندازه کوچک، سطح مقطع زیاد، شکل کریستالی و نظم شبکه‌ای منحصر به فرد و در نتیجه واکنش پذیری بسیار زیاد و عملکرد قابل توجه نانوتیوب‌های کربنی چند‌جداره به عنوان جاذب در حذف آلاینده‌های آلی از محلول‌های آب، این جاذب می‌تواند جهت حذف اسید هیومیک مؤثر باشد.

Title: کارایی راکتورهای بیوفیلمی بستر متحرک حاوی کالدنس نوع 1 در حذف ترکیبات آلی و ازته تحت شرایط محدود اکسیژن محلول از فاضلاب

http://tbj.ssu.ac.ir/article-1-329-fa.html

سابقه و اهداف: یکی از سیستمهای بیولوژیکی ترکیبی نوین که در تصفیه فاضلاب حاوی مواد آلی و ازت بکار برده می شود، سیستم راکتورهای بیولوژیکی با بستر متحرک ( Moving Bed Biofilm Reactors - MBBR ) است. در این تحقیق، این فرایند در راکتورهای بیوفیلمی با بستر متحرک حاوی آکنه های KMT ( k1 ) در مقیاس آزمایشگاهی مورد بررسی قرار گرفت.   روش بررسی: در این سیستم، از دو راکتور سری بصورت انوکسیک ( R1 ) و هوازی ( R2 ) بترتیب دارای حجم 5/3 و 10 لیتر که در دمای oC 5/28 ، 8- 7، pH = ، mg/l 1- 5/1 DO = و نسبت برگشت داخلی Q 3 بصورت پیوسته استفاده شد. کلیه آزمایشات بر اساس کتاب ستاندارد متد انجام شد. نسبت پر شدگی راکتورهای R1 و R2 با آکنه های k1 به ترتیب 40 و 50 در صد حجمی بود. آنالیز آماری داده ها با نرم افزار spss و excel انجام شد.   یافته ها: متوسط غلظت اکسیژن محلول در راکتور هوازی در دوره بهره بردازی در دامنه mg/l 5/1-1 بود. متوسط راندمان حذف کربن آلی ( SCOD ) در بارگذاریهای مختلف ( mg/l 2000-300) بیش از 95% بود و بیشترین میزان آن در راکتور انوکسیک حذف شد. راندمان حذف نینروژن در بارگذاری های مختلف ( mg -N/l 250-25) در دامنه 4/99-80 درصد متغییر بود و بیشترین راندمان حذف در بارگذاری mg/m 2 . day 181/0-127/0 براساس سطح بیوفیلم در راکتور انوکسیک در حضور کربن آلی (حدود 92%) بدست آمد. نتایج نشان داد که با افزایش میزان بارگذاری ترکیبات ازته، نیتریفیکاسیون افزایش یافته، اما سرعت افزایش آن در بارگذاریهای بالا، کاهش یافته است. علت آن به احتمال زیاد تجمع یون نیتریت در راکتور هوازی می باشد.   نتیجه گیری: نتایج نشان داد که فرایند حذف ترکیبات آلی و ازته از فاضلاب در سیستم MBBR به روش پیش دنیتریفیکاسیون، بدون برگشت لجن و بدون استفاده از منبع کربن خارجی، در شرایط محدود اکسیژن محلول در راکتور هوازی، دارای عملکرد قابل توجهی جهت تصفیه فاضلاب های دارای مقادیر زیاد ترکیبات ازته و آلی می باشد.    ( k1 )

Title: شناسایی خطرات و ارزیابی ریسک فرآیند تونل سازی با بکارگیری از تکنیک آنالیز ایمنی شغل

http://tbj.ssu.ac.ir/article-1-330-fa.html

سابقه و اهداف: بدون تردید یکی از عوامل تأثیر گذار بر سلامت شاغلین پروژه های عمرانی مانند تونلسازی، خطرات بالقوه در حین اجرا می باشد که می­تواند خسارات جبران ناپذیری را ببار ­آورد. بنابراین شناسایی خطرات و ارزیابی ریسک فرایند تونلسازی با بکارگیری از تکنیک آنالیز ایمنی شغلی در این مطالعه مد نظر می باشد.   روش بررسی: مطالعه توصیفی حاضر خطرات بالقوه فعالیتهای تونلسازی یکی از پروژه های بزرگ عمرانی سد و نیروگاه آبی در حال ساخت را با تکنیک آنالیز ایمنی شغلی Job Safety Analysis) ) را شناسایی و سپس سطح ریسک فعالیتها ارزیابی گردیده است. اجرای آنالیز ایمنی شغلی، طبق روش توصیه شده در استاندارد OSHA 3071 و تعیین سطح ریسک های موجود طبق استاندارد MIL-STD-882E (ویرایش پنجم) انجام گرفته است. سطح ریسک هر فعالیت بر اساس چهار شاخص قضاوت شامل شدید، نسبتاً شدید، متوسط و پایین ارائه گردیده است.   یافته ها: در مجموع 232 خطربالقوه شناسایی گردید. نتایج نشان می­دهد 9/15% درصد از ریسکهای شناسایی شده در منطقه شدید، 3/17% نسبتاً شدید، 4/38% متوسط و 4/28% در محدوده پائین قرار داشتند. بیش از یک سوم خطرات شناسایی در محدوده غیر قابل قبول قرار گرفته اند که علت عمده آن را می توان انجام کار در ارتفاع بدون استفاده از تجهیزات مناسب، تأخیر در تحکیمات سنگهای ناپایدار و کمبود تجهیزات رفتار شناسی تونل برشمرد.   نتیجه گیری: انتظار می رود با تأمین اعتبار مالی، فراهم نمودن تجهیزات و ابزار کار و نظارت بر انجام کار به روش ایمن بتوان سطح ریسک های موجود را بطور چشمگیری کاهش داد.

Title: مقایسه اثر مصرف چای سبز و ترش بر قندو پرو فایل چربی در بیماران مبتلا به دیابت

http://tbj.ssu.ac.ir/article-1-331-fa.html

سابقه و اهداف: دیابت ملیتوس یکی از بیماری های شایع غدد درون ریز می باشد که به دلیل توسعه زندگی ماشینی بروز آن در اکثر نقاط دنیا روز به روز در حال افزایش است. اختلال چربی ها یکی از اختلالات متداول در این بیماری است که خود سبب پیدایش و تشدید عوارض کوتاه و بلند مدت آن می شود. استفاده از گیاهان دارویی از جمله استراتژی های درمانی برای پیشگیری و کنترل عوارض ناشی از بیماری دیابت می باشد که نمونه هایی از آنها گیاه چای ترش و سبز است. مطالعه حاضر با هدف مقایسه اثر مصرف چای ترش و سبز بر قند و چربی بیماران دیابت نوع دو دارای اضافه وزن و چاقی انجام شده است.   روش بررسی : این مطالعه یک کارآزمایی بالینی تصادفی می باشد که با مشارکت 100 بیمار مبتلا به دیابت تحت پوشش مرکز تحقیقات یزد در طول سال 1390 انجام گرفت. افراد به طور تصادفی به دو گروه چای ترش و چای سبز تقسیم شدند. هر دو گروه به مدت 4 هفته روزانه سه بار و هر بار 150 سی سی به فاصله 2 ساعت بعد از هر وعده ی غذایی چای مربوط به خود را مصرف کردند. قند ناشتا و پروفایل لیپیدی در ابتدا و انتهای مداخله اندازه گیری شد.   یافته ها : نود و چهار نفر مطالعه را به پایان بردند. میانگین متغیرهای دموگرافیک، قند و چربی ها در آغاز و پایان مداخله بین دو گروه تفاوت معنی داری نشان نداد. در هر دو گروه تنها HDL-C در پایان نسبت به آغاز مداخله افزایش معنی داری داشت، به طوری که در گروه گیرنده چای ترش میانگین آن از 4/8 ± 1/37 آغاز مطالعه به 8/8 ± 8/41 میلی گرم در دسی لیتر (6/16 درصد افزایش) و در گروه گیرنده چای سبز از 1/9 ± 7/38 به 13 ± 43 میلی گرم در دسی لیتر (14 درصد افزایش ) رسیده است.   نتیجه گیری: مطالعه حاضر نشان داد که مصرف چای ترش و سبز هر دو بر افزایش معنی دار HDL-C موثرند. بنابراین با عنایت به این اثر و سایر اثرات مفید گزارش شده از مطالعات مشابه، نوشیدن آنها در بیماران دیس لیپیدمیک به ویژه بیماران مبتلا به دیابت قابل توصیه است.

Title: آگاهی، نگرش و عملکرد مردم شهر یزد درباره سرطان پوست

http://tbj.ssu.ac.ir/article-1-332-fa.html

سابقه و هدف: سرطان پوست، شامل سرطان پوست غیر ملانوم ( NMSC ) و ملانوم، فراوانترین سرطان در بین سرطانها می باشد. در ایران نیز شایعترین سرطان، سرطان پوست است. هدف این مطالعه بررسی آگاهی، نگرش و عملکرد مردم شهر یزد درباره سرطان پوست و محافظت در برابر آفتاب بود.   روش بررسی: پژوهش حاضر مطالعه ای توصیفی- تحلیلی از نوع مقطعی می باشد. شرکت کنندگان 360 زن و مرد در فاصله سنی 55-18 سال، در شهر یزد بودند. داده ها به کمک یک پرسشنامه خود گزارشی جمع آوری شد. سپس داده ها از طریق نرم افزار آماری SPSS با استفاده از آزمون های آماری، T- test ، ANOVA ، Kruskal-Wallis ، Chi-Square ، Pearson Correlation ، Spearman Correlation مورد تجزیه و تحلیل قرار گرفت.   یافته ها: از 358 شرکت کننده که پرسشنامه ها را تکمیل کردند، 44 ٪ مرد و 56 ٪ زن بودند. میانگین نمرات آگاهی 04/5 ± 72/12 ، نگرش 95/2 ± 94/28 و عملکرد 75/1 ± 37/8 بود. این مطالعه نشان داد آگاهی ارتباط معنی داری با سن، جنس، تحصیلام و شغل(05/0 P < ). نگرش نسبت به سرطان پوست ارتباط معنی داری با تحصیلات و شغل داشت(05/0 P < ). رفتار ارتباط معنی داری با تحصیلات داشت(05/0 P < ). همچنین ارتباط معنی داری بین آگاهی درباره سرطان پوست با نگرش و عملکرد و بین نگرش با عملکرد وجود داشت.   نتیجه گیری: این مطالعه تاکید دارد بر اهمیت نقش آگاهی در نگرش و رفتار و نقش نگرش در رفتار. همچنین سطح آگاهی، نگرش و عملکرد مردم تحت تاثیر چندین فاکتور شامل جنس، سن، تحصیلات و شغل است. این فاکتورها باید به هنگام طراحی برنامه های مداخله ای درباره سرطان پوست و محافظت در برابر آن در نظر گرفته شود.
